# Interleukin 12 receptor deficiency in a child with recurrent bronchopneumonia and very high IgE levels

**DOI:** 10.1186/1824-7288-38-46

**Published:** 2012-09-19

**Authors:** Loredana Palamaro, Giuliana Giardino, Francesca Santamaria, Rosa Romano, Anna Fusco, Silvia Montella, Mariacarolina Salerno, Matilde Valeria Ursini, Claudio Pignata

**Affiliations:** 1 Department of Pediatrics, “Federico II” University, Naples, Italy; 2 International Institute of Genetics and Biophysics, CNR, Naples, Italy; 3 Unit of Immunology, Department of Pediatrics, “Federico II” University, Via S. Pansini 5-80131, Naples, 80127, Italy

**Keywords:** Immunodeficiency, IL-12/IL-12 receptor, Recurrent pneumonia

## Abstract

Interleukin-12 (IL-12) is involved in cellular immune responses against intracellular pathogens by promoting the generation of T naive in T helper 1 (Th1) cells and by increasing interferon-gamma (IFN-gamma) production from T and natural killer (NK) cells. A defective induction of a Th1 response may lead to a higher risk of infections, and, in particular, infections due to typical and atypical *Mycobacteria*. We report on the case of a girl with suffering from recurrent bronchopneumonia associated with very high serum IgE levels, who exhibited a profound impairment of the Th1 generation associated with a novel mutation in the exon 5 of the IL-12R β1 gene (R156H). Our data suggest that in children with severe and recurrent infections, even in the absence of a mycobacterial infection, functional and/or genetic alterations of the molecular mechanisms governing Th1/Th2 homeostasis might be responsible for an atypical immunodeficiency and, therefore, should be investigated in these patients.

## Background

Primary congenital immunodeficiencies encompass a wide spectrum of distinct clinical entities, which differ in either pathogenetic mechanism or clinical features. Recently, several novel syndromes with unusual phenotypes have been described [1,2]. However, in a number of patients suffering from severe and sometimes life-threatening infections, in which an immunological disorder is suspected, the underlying genetic defect responsible for the immunodeficiency still remains to be elucidated [3]. Recently, a higher susceptibility to intracellular pathogens and, in particular, atypical mycobacterial and salmonella infections has been described in patients with genetic alterations of the IL-12 receptor (IL-12R) [4-9]. IL-12 stimulates cellular immune responses against intracellular pathogens by promoting the generation of T naive in T helper 1 (Th1) cells and by increasing interferon-gamma (IFN-gamma) production from T and natural killer (NK) cells. Induction of a Th1 response and cell cycle progression mostly relies on the expression of a high affinity IL-12R, consisting of β1 and β2 chains [10-15]. A few genetic alterations of β1 chain have already been reported in patients suffering of mycobacterial infections [5,6].

## Case presentation

A 8-year-old girl was referred to the our Department because of a history of recurrent pneumonia (4 episodes over 2 years). At the age of 4 years and 8 months she had suffered from the first episode of middle lobar bronchopneumonia requiring hospitalization. In that occasion the total IgE serum levels were 3350 kU/l. One month later, she was hospitalized for a second bronchopneumonia episode interesting both lungs followed by persistent cough for more than a month. These episodes were responsive to antibiotic therapy. Subsequently, she had suffered from 2 additional bronchopneumonia episodes in distinct lung area, successfully treated with parenteral antibiotic therapy. Conventional x-ray and high resolution computed tomography of the chest revealed multiple focal consolidations in both lungs, confirmed by magnetic resonance imaging [16]. Acid resistant bacillus was not found in the sputum examination. In one occasion, *Haemophilus influenzae* was isolated on sputum culture. Weight and height growth was in the normal range. No infections in other organs were reported. The routine immunological evaluation revealed normal IgG and IgA, but very high serum IgE levels (> 2000 kU/l), confirmed in several occasions during the 2 years follow-up. Specific IgE toward Dermatophagoides farinae and pteronyss, olive, herb vitriol and Parietaria judaica were detected. Prick test were positive (ponf > 0.3 × 0.4 cm) for Dermatophagoides farinae and pteronyss, hair of dog, Parietaria and olive, thus confirming a multiple sensitivity. The patient showed a proper antibody specific response as demonstrated by the presence of IgG antibody serum levels, tested by immuno-enzymatic method, against B-hepatitis, parotitis and German measles viruses. Serum IgG, IgA e IgM levels were always in the normal range. The immunophenotype valuation revealed normal number and percentage of the lymphocyte subpopulations studied (Table 1).

**Table 1:** Immunonological parameters

**Lymphocyte subpopulations**	**%**	**n/mm**^**3**^
CD3	77	2.956
CD3DR	4.2	161.28
CD4	39.3	1509
CD8	30.2	1159
CD19	11.4	437.76
CD56	3.4	130.56
CD4-CD8-TCR α/β+	2.3	88.32
CD4-CD8-TCR g/d+	2.2	84.48
**Specific antibody responses**	**IgG**	**IgM**
B-hepatitis virus	Present	Absent
Parotidis virus	Present	Absent
German measles virus	Present	Absent
**Proliferative response to mitogens stimulation**	**Patient (mean ± SE)**	**Control (mean ± SE)**
PMA + iono	22458 ± 11013	32159 ± 27858
CD3 X-L	800 ± 68	29500 ± 3000

The patient’s family history was notable for the presence of allergic disorders in both lineages. In particular, her mother and grandmother had a history of allergic rhinitis, while her father had urticaria. A maternal aunt died at 2 years of age by whooping-cough and a maternal uncle died at 16 months by a severe not better specified respiratory infection. A paternal aunt and her daughter had a history of allergic rhinitis.

**Figure 1:**
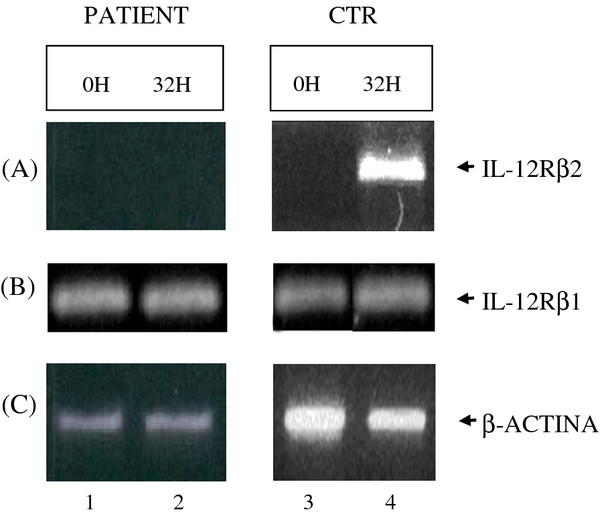
**mRNA expression of IL-12R β2 chain after mitogen stimulation of PBMC in vitro.** (**A**) No expression of IL-12R β2 transcript was found in patient’s cells, differently from the controls. (**B**) IL-12R β1 was expressed at normal levels.

We first determined the proliferative response of PBMC to CD3 cross-linking, that mimics in vivo antigen exposure, performed at optimal (1 ng/ml) or suboptimal (0.3 ng/ml) antibody concentration. The proliferation at the maximal dosage was significantly lower in the patient than in the controls (mean ± SE were: 800 ± 68 cpm and 29500 ± 3000 cpm in the proband and controls, respectively). Since a proper immune response to pathogens requires a Th1 induction and this process determines up-regulation of the expression of the IL-12R β2 chain, we evaluated mRNA expression of this molecule after mitogen stimulation of PBMC in vitro. No expression of IL-12R β2 transcript was found in patient’s cells (Figure 1A), differently from the controls. IL-12R β1 was expressed at normal levels (Figure 1B).

**Figure 2:**
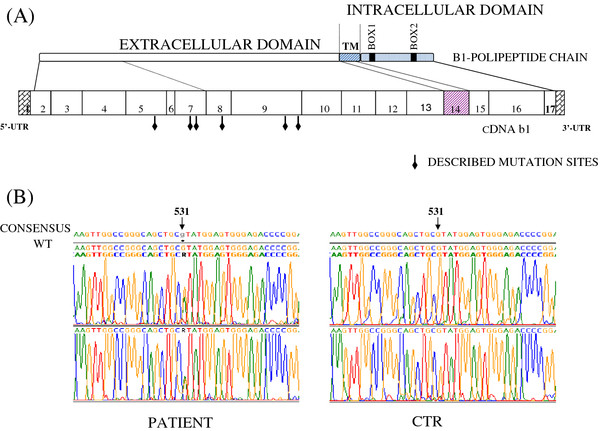
**gene sequencing of IL-12R β2 gene.** At molecular level, gene sequencing of IL-12R β2 gene revealed a missense mutation (G to A) at nucleotide 531 in the exon 5 in heterozygosity, resulting in the substitution of arginine **(**CGT) with histidine (CAT) in the extracellular domain of the receptor at the same aminoacid position 156 (designed R156H).

At molecular level, gene sequencing of IL-12R β2 gene revealed a missense mutation (G to A) at nucleotide 531 in the exon 5 in heterozygosity, resulting in the substitution of arginine (CGT) with histidine (CAT) in the extracellular domain of the receptor at the same aminoacid position 156 (designed R156H) (Figure 2). The mutation was not a polymorphism since was not found in 100 chromosomes from unrelated individuals. This G to A transition creates a new restriction site for NdeI enzyme (data not shown).

## Discussion

The case here reported indicates that alterations of the induction of a proper Th1 response may be associated with an atypical immunodeficiency characterized by high susceptibility to infections. The functional response of lymphocytes to IL-12 depends on the expression of a high affinity IL-12 receptor on Th1 and NK cells. The high affinity receptor for IL-12 consists of two subunits, β1 and β2, closely related to the cytokine receptor glycoprotein (gp) 130 [11,17]. The complete IL-12R is thought to be associated with the development, being expressed on human naive T cells during differentiation to Th1 but not to Th2. Therefore, the expression of these molecules is generally considered as a marker of Th1 dominated response [11-13,16]. Th1 cells produce IFN-gamma and IL-2 and, predominantly, promote cell mediate immune responses against intracellular pathogens [18,19]. In a previous study, we provided evidence of altered IL-12/IL-12R signaling in patients with very high IgE levels, suggestive of an impaired Th1 induction [20]. A defective induction of a Th1 response in patients may lead to a higher risk of infections, thus worsening the overall outcome of patients with very high IgE levels. In the case herein described a genetic alteration of the IL-12R β1 has been found in heterozygosity. Whether this alteration is really responsible for the phenotype remains to be definitively demonstrated with further molecular and functional studies. However, it should be noted that patients with homozygous alterations of the same gene have already been reported, being affected with a more severe clinical phenotype and selective susceptibility to mycobacterial infections [5,6]. Based on this clinical observation, we suggest that a better understanding of the molecular mechanisms governing Th1/Th2 homeostasis may help recognize novel clinical phenotypes of atypical immunodeficiencies.

## Consent

Written informed consent was obtained from the parents of the patient for publication of this Case report and any accompanying images.

## Abbreviations

IL-12: Interleukin-12; Th1: T helper 1; IFN-gamma: Interferon-gamma; NK: Natural Killer.

## Competing interests

The authors declare that they have no competing interests.

## Authors’ contributions

LP has made substantial contributions to conception and design, has been involved in drafting the manuscript, and has given final approval of the version to be published. GG has made substantial contributions to conception and design, has been involved in drafting the manuscript, and has given final approval of the version to be published. FS has made substantial contributions to conception and design, has been involved in revising the manuscript critically for important intellectual content, and has given final approval of the version to be published. RR and AF has made substantial contributions to acquisition of data, has been involved in drafting the manuscript, and has given final approval of the version to be published. SM has made substantial contributions to acquisition of data, has been involved in revising the manuscript critically for important intellectual content, and has given final approval of the version to be published.

MVU has made substantial contributions to conception and design and analysis and interpretation of data, has been involved in revising the manuscript critically for important intellectual content, and has given final approval of the version to be published. CP has made substantial contributions to conception and design and analysis and interpretation of data, has been involved in drafting the manuscript and revising it critically for important intellectual content, and has given final approval of the version to be published.
